# Convolutional Neural Network–Based Prediction of Axial Length Using Color Fundus Photography

**DOI:** 10.1167/tvst.13.5.23

**Published:** 2024-05-29

**Authors:** Che-Ning Yang, Wei-Li Chen, Hsu-Hang Yeh, Hsiao-Sang Chu, Jo-Hsuan Wu, Yi-Ting Hsieh

**Affiliations:** 1 School of Medicine, National Taiwan University, Taipei, Taiwan; 2 Department of Ophthalmology, National Taiwan University Hospital, Taipei, Taiwan; 3 Graduate Institute of Clinical Medicine, College of Medicine, National Taiwan University, Taipei, Taiwan; 4 Shiley Eye Institute and Viterbi Family Department of Ophthalmology, University of California San Diego, La Jolla, CA, USA

**Keywords:** axial length, color fundus; machine learning, CNN, age

## Abstract

**Purpose:**

To develop convolutional neural network (CNN)–based models for predicting the axial length (AL) using color fundus photography (CFP) and explore associated clinical and structural characteristics.

**Methods:**

This study enrolled 1105 fundus images from 467 participants with ALs ranging from 19.91 to 32.59 mm, obtained at National Taiwan University Hospital between 2020 and 2021. The AL measurements obtained from a scanning laser interferometer served as the gold standard. The accuracy of prediction was compared among CNN-based models with different inputs, including CFP, age, and/or sex. Heatmaps were interpreted by integrated gradients.

**Results:**

Using age, sex, and CFP as input, the mean ± standard deviation absolute error (MAE) for AL prediction by the model was 0.771 ± 0.128 mm, outperforming models that used age and sex alone (1.263 ± 0.115 mm; *P* < 0.001) and CFP alone (0.831 ± 0.216 mm; *P* = 0.016) by 39.0% and 7.31%, respectively. The removal of relatively poor-quality CFPs resulted in a slight MAE reduction to 0.759 ± 0.120 mm without statistical significance (*P* = 0.24). The inclusion of age and CFP improved prediction accuracy by 5.59% (*P* = 0.043), while adding sex had no significant improvement (*P* = 0.41). The optic disc and temporal peripapillary area were highlighted as the focused areas on the heatmaps.

**Conclusions:**

Deep learning–based prediction of AL using CFP was fairly accurate and enhanced by age inclusion. The optic disc and temporal peripapillary area may contain crucial structural information for AL prediction in CFP.

**Translational Relevance:**

This study might aid AL assessments and the understanding of the morphologic characteristics of the fundus related to AL.

## Introduction

Axial length (AL) is an important parameter in ophthalmology. Notably, the progression of myopia is predominantly attributable to AL elongation.^1^^,^^2^ Furthermore, the process of AL elongation leads to the shift of refractive error and causes morphologic changes in the fundus. In long eyes, distinct fundus anomalies such as fundus tessellation, peripapillary atrophy (PPA), and diffuse or patchy chorioretinal atrophy can often be observed.^3^ Additionally, some structural configurations have been reported to be closely associated with a long AL. These configurations encompass elongation of the disc–fovea distance, enlargement of the gamma zone, reduction in angle κ, and straightening of the papillomacular retinal vessel.^4^^,^^5^ Notably, these retinal and choroidal characteristics associated with AL elongation can be effectively observed using color fundus photography (CFP). Based on the correlative relationship between fundus appearances and AL, Dong et al.^6^ developed deep learning–based models utilizing CFP to predict AL with a rather good accuracy.

However, AL is not the sole determining factor in the morphologic aspects of the fundus; sex and age have been reported as related factors. Yamashita et al.^7^ discerned significant gender-based disparities in retinal vessel angles and tessellation within the peripapillary region. Furthermore, deep learning–based models using CFP for distinguishing between sexes were developed,^8^ which provided an example showing that artificial intelligence is capable of detecting sexual distinctions in fundus features. Regarding age, several studies^9^^,^^10^ have expounded that a significant correlation exists between aging and retinal vascular structures, thereby endowing these structures with the potential to serve as an indicator of one’s cardiovascular health. Moreover, the color content of fundus images was found to be shifted during aging.^11^ Remarkably, both retinal vascular structures and color content are included in CFP. Due to the age-related changes of CFP, Wang et al.^12^ developed convolutional neural network (CNN) models by integrating retinal fundus and vessel images as combined data inputs for predicting age, and the results showed commendable performance.

Recently, deep learning techniques have been widely applied in the domain of medical image analysis, especially in the field of ophthalmology.^13^ Notably, the performance of deep learning for fundus image diagnostic tasks is quite impressive and has achieved better performance than human experts in some cases.^14^^,^^15^ In this study, we primarily aimed to develop CNN-based models for predicting AL using CFP and structural characteristics, including age and/or sex, and the result was used to explore the correlation of AL-, sex-, and age-related features to provide a new understanding of the fundus structure. Particularly, the principal intent of this study was not to replace the traditional way of AL measurement via optical biometry. However, it can provide an alternative AL measuring method with great accuracy. For example, in cases where cataract surgery is only found to be needed during pars plana vitrectomy, AL measurement via optical biometry would be difficult, and it would be more convenient to estimate the AL using CFP.

## Methods

### Data Collection and Preprocessing

Patients who underwent CFP and AL examinations for the preoperative evaluation for cataract surgery at National Taiwan University Hospital from 2020 to 2021 were retrospectively recruited. Exclusion criteria were any retinal or choroidal diseases that can be observed in the CFP and those with severe cataracts, which make the optic disc completely invisible. Only images that clearly showed the optic disc and the central fovea were included. Finally, a collection of 1105 color fundus images of 864 eyes from 467 patients was enrolled. In the 1105 images, macular-centered images of all eyes were included, while there were additional optic disc–centered images for some eyes. Moreover, to check the effect of the quality of the CFP, a subanalysis was performed, specifically focusing on high-quality CFP (HQ-CFP). HQ-CFP was defined as CFP showing clear retinal vessels in which venula macularis superior, arteriola macularis superior, arteriola macularis inferior, and venula macularis inferior could be distinguished. This subset of HQ-CFP comprised 918 images of 750 eyes from 409 patients. Afterward, we extracted the age, sex, and AL data of these patients from the preoperative medical records. The Lenstar LS 700 optical biometer (Haag-Streit AG, Köniz, Switzerland) was used for all ALs data measurements. This study was approved by the Institutional Review Board of National Taiwan University Hospital (202110087RINC).

Our model employed two input sources: structured data (age and sex) and CFP. We encoded sex as a binary variable and standardized the age variable by subtraction of the mean and division by the standard deviation (SD). As for the CFPs, we preprocessed the images, which were originally in 2736 × 1824 resolution, by cropping 1640 × 1640 pixels at the center and resizing the images to a lower resolution of 300 × 300 for model input. No additional image preprocessing was made. Different compositions of predictors were placed in each model to test the efficacy of these factors, and six models were built. Model l contained structured data (age and sex), while model 2 included CFP only. Models 3 to 5 were the different combinations of structured data and CFP, where model 3 included CFP and age, model 4 included CFP and sex, and model 5 included CFP, age, and sex. Model 6 had inputs similar to those of model 5, and the only difference was that HQ-CFP was used instead of CFP.

### Model Development

**Figure 1.**
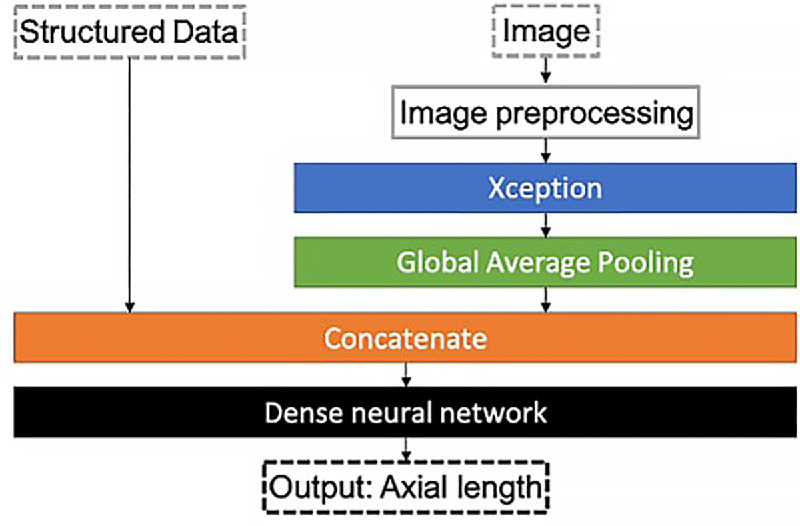
The process for model development. Image input was pretrained by the deep learning model “Xception” after preprocessing. The model was then trained by a Nadam optimizer with a learning rate of 0.001 in the first 10 epochs, 0.0005 for the next 10 epochs, and an exponential decay of 0.9 for every 5 epochs afterward.

The architecture for model development is illustrated in Figure 1. Image input was first processed by a pretrained deep learning model called Xception^16^ for feature extraction; it was pooled by global average pooling, and the batch was normalized before it was concatenated with age and/or sex. The combined information went through a dense layer with a one-dimensional output for the final AL prediction. We used the weights of Xception trained on Imagenet^17^ and removed the top layers of Xception to keep only the feature extraction part for our model. Consequently, we conducted a series of iterative training and weight updates using the CFP, accompanied by the actual AL, which allowed us to fine-tune the initial weights progressively. The model was built with TensorFlow 2.4.0 and Python 3.8 using the Keras library.

We used the root-mean-square loss function to optimize the model. The model was trained by a Nadam optimizer^18^ with a learning rate of 0.001 in the first 10 epochs, 0.0005 for the next 10 epochs, and an exponential decay of 0.9 for every 5 epochs afterward. Early stopping was applied if the validation loss did not improve for 25 epochs.

### Model Validation and Heatmap Generation

To efficiently use the relatively small data set and generate robust estimates for test errors, we used nested 10-fold cross-validation to generate 100 test results for model evaluation. We divided the whole data set into 10 nonoverlapping test sets in the first layer; for each test set, we subdivided the remaining 90% of data into 10 different sets of validation data in the second layer. For each pair of test and validation data, the remaining data that were not selected (roughly 81% of the original data) were used for training. Besides, no test and validation data set would contain any CFP from patients whose CFP was also present in the training data set. This process generated 100 different combinations of training, validation, and test sets that allowed us to estimate the distribution of the test errors and the 95% confidence interval. Tenfold validation was implemented 100 times, and the 100 mean absolute errors (MAEs) were taken out on average, illustrating the accuracy of the model's prediction of AL. We also qualitatively evaluated the models by visualizing crucial areas for prediction in the test images with the integrated gradient method. The gradients were computed for the output of the highest-scoring class regarding the pixel of the input image and can be visualized by aggregating them along the color channel and scaling the pixels.^19^ Afterward, the plotted gradients were overlayered onto the image for visual comparison.

### Statistical Analysis

Scanning laser interferometer was considered the gold standard; thus, the estimation error of AL was defined as the difference between interferometer measurements and deep learning estimation. The estimation errors were used to calculate the MAE to determine the efficacy of the predictors in the deep learning models. Single-tailed independent sample *t*-tests were performed to check the statistical difference of the average MAEs between models. STATA (V.14; Stata Corp LP., College Station, TX, USA) was used for analysis, and a *P* < 0.05 was considered statistically significant.

## Results

### Data Characteristics

This study included 1105 fundus images from 864 eyes of 467 participants. Of the 467 participants, 171 (36.6%) were male, with a mean ± SD age of 69.64 ± 9.97 years (range, 39.67–92.75 years). Of the 864 eyes, 241 had macular- and optic disc–centered images included. The mean ± SD AL of the included eyes was 24.80 ± 1.99 mm (range, 19.91–32.59 mm). Table 1 details the characteristics of the eyes. In total, 918 images of 750 eyes from 409 patients were eventually included in the set of HQ-CFP for the subanalysis of CFP quality control, while the range of AL remained 19.91 to 32.59 mm. A total of 16.92% of images of relatively poor quality were removed.

**Table 1.** Baseline Characteristics of the Data Set

Characteristic	Value
Number of participants	467 (864 eyes)
Number of images	1105
Axial length, mean ± SD, mm	24.80 ± 1.99
Minimum and maximum, mm	19.91, 32.59
<22 mm	17 (1.97%)
≥22 mm and <26 mm	638 (73.8%)
≥26 mm	209 (24.2%)
Age, mean ± SD, y	69.64 ± 9.97
Minimum and maximum, y	39.67, 92.75
Sex, male, %	36.6

### Models for Predicting the AL With Different Input Parameters

The MAEs of all models with different input parameters (CFP, age, and/or sex) are shown as models 1 to 6 in Table 2. Compared to model 1, which included only age and sex as the predictors (mean ± SD MAE = 1.263 ± 0.115 mm), model 2, which used CFP as the only input, had a 34.20% decrease in MAE (0.831 ± 0.216 mm) for AL prediction (*P* = 0.043). Compared to model 2, including age in addition to CFP as the predictor in model 3 further decreased the MAE by –5.59% (*P* = 0.043). In contrast, the addition of sex to CFP as the predictor in model 4 did not significantly decrease the MAE (*P* = 0.405). From Table 3, the effect of adding age or sex as an additional predictor was clearly demonstrated. No matter the composition of the original predictor(s), adding age to the model could improve the performance significantly (*P* = 0.043, 0.008), while adding sex to the model did not make obvious changes (*P* = 0.41, 0.24).

**Table 2.** Prediction Models for the Axial Length With Various Composition of Input Factors and Their Validated Prediction Errors

Model	Input Parameters Selected	MAE (SD), mm	Comparison With Model 1, %	*P* Value	Comparison With Model 2, %	*P* Value	Comparison With Model 5, %	*P* Value
Model l	Age and sex	1.263 (0.115)	Reference	—	—	—	—	—
Model 2	CFP	0.831 (0.216)	−34.20	<0.001	Reference	—	—	—
Model 3	CFP and age	0.785 (0.161)	−37.88	<0.001	−5.59	0.043	—	—
Model 4	CFP and sex	0.825 (0.182)	−34.73	<0.001	−0.72	0.41	—	—
Model 5	CFP, age, and sex	0.771 (0.128)	−39.01	<0.001	−7.31	0.008	Reference	—
Model 6	HQ-CFP, age, and sex	0.759 (0.120)	−39.90	<0.001	−9.49	0.003	−1.56	0.24

**Table 3.** Improvements in Predicting the Axial Length With Color Fundus Photography by Adding Age or Sex as the Additional Predictor

Additional Predictor	Original Predictor(s)	Change in MAE, %	*P* Value
Age	CFP	−5.59	0.043
	CFP, sex	−6.55	0.008
Sex	CFP	−0.72	0.41
	CFP, age	−1.78	0.24

**Figure 2.**
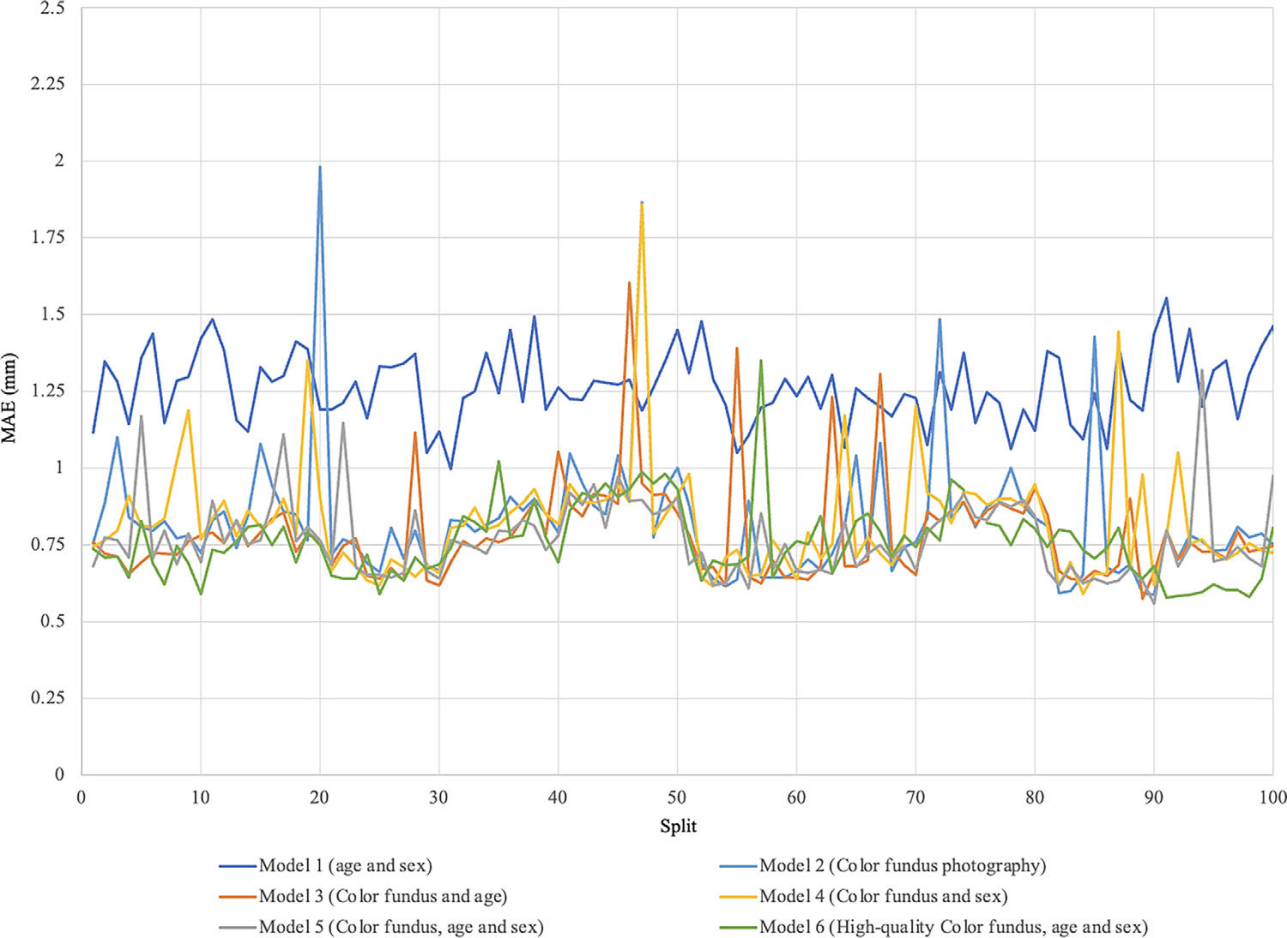
The 100 mean absolute errors for 10-fold validation of each axial length prediction model.

**Figure 3.**
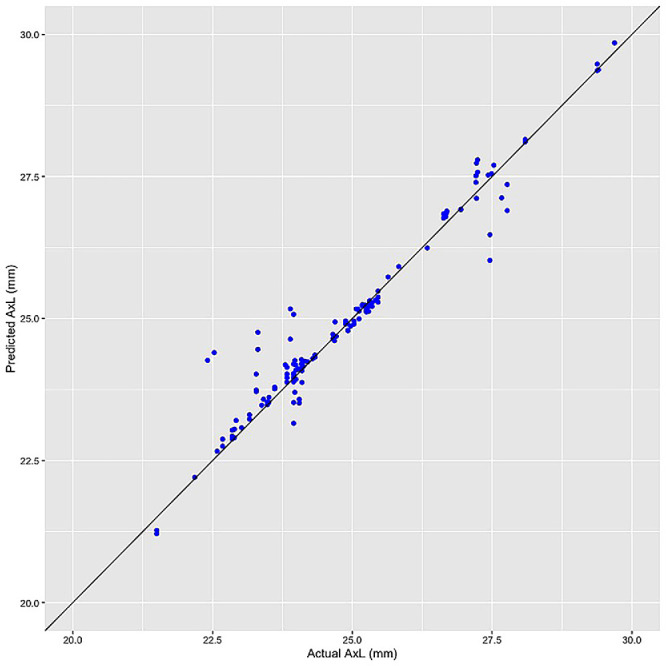
The distribution of predicted and actual AL in a test of model 5 (CFP, age, and sex). Eyes with long ALs tended to be underestimated, while those with short ALs tended to be overestimated.

Figure 2 shows the overall comparison of MAEs across different models**.** Under the same set of CFP, the smallest MAE (0.771 ± 0.128 mm) was achieved by model 5, which used CFP, age, and sex as the predictors. As shown in Figure 3, the models tended to underestimate AL in eyes with longer ALs and overestimate AL in eyes with shorter ALs. In addition, the comparison of models 5 and 6 was used to test whether removing relatively poor-quality images made the model perform better, as shown in Table 2. Under the same input parameters, using the image set of HQ-CFP to replace CFP further decreased the MAE by −1.56% to 0.759 ± 0.120 mm, but there was no statistically significant difference (*P* = 0.237).

### Heatmaps of Color Fundus Photography for AL Prediction

Figure 4 shows some examples of heatmap analysis generated by integrated gradients, which indicates the CFP areas most highlighted by the deep learning models during AL prediction. Well and poor prediction samples in both the best and the worst split in model 5 (input predictors: sex, age, and CFP) and model 6 (input predictors: sex, age, and HQ-CFP) are shown. The optic disc and the peripapillary area, especially the temporal peripapillary area, were most frequently highlighted on the heatmaps of examples with good model prediction, regardless of the AL measurement. As the AL became longer, the temporal PPA and disc tilting were more significantly highlighted, while almost no PPA was observed in shorter eyes. Tessellated choroidal vessels were also highlighted in some cases. The characteristics mentioned above could be observed regardless of the best or the worst prediction split between both models. As for the heatmaps of examples of poor model prediction (absolute error >0.5 mm), the optic disc and peripapillary area were still the most highlighted, followed by the tessellated choroidal vessels.

**Figure 4.**
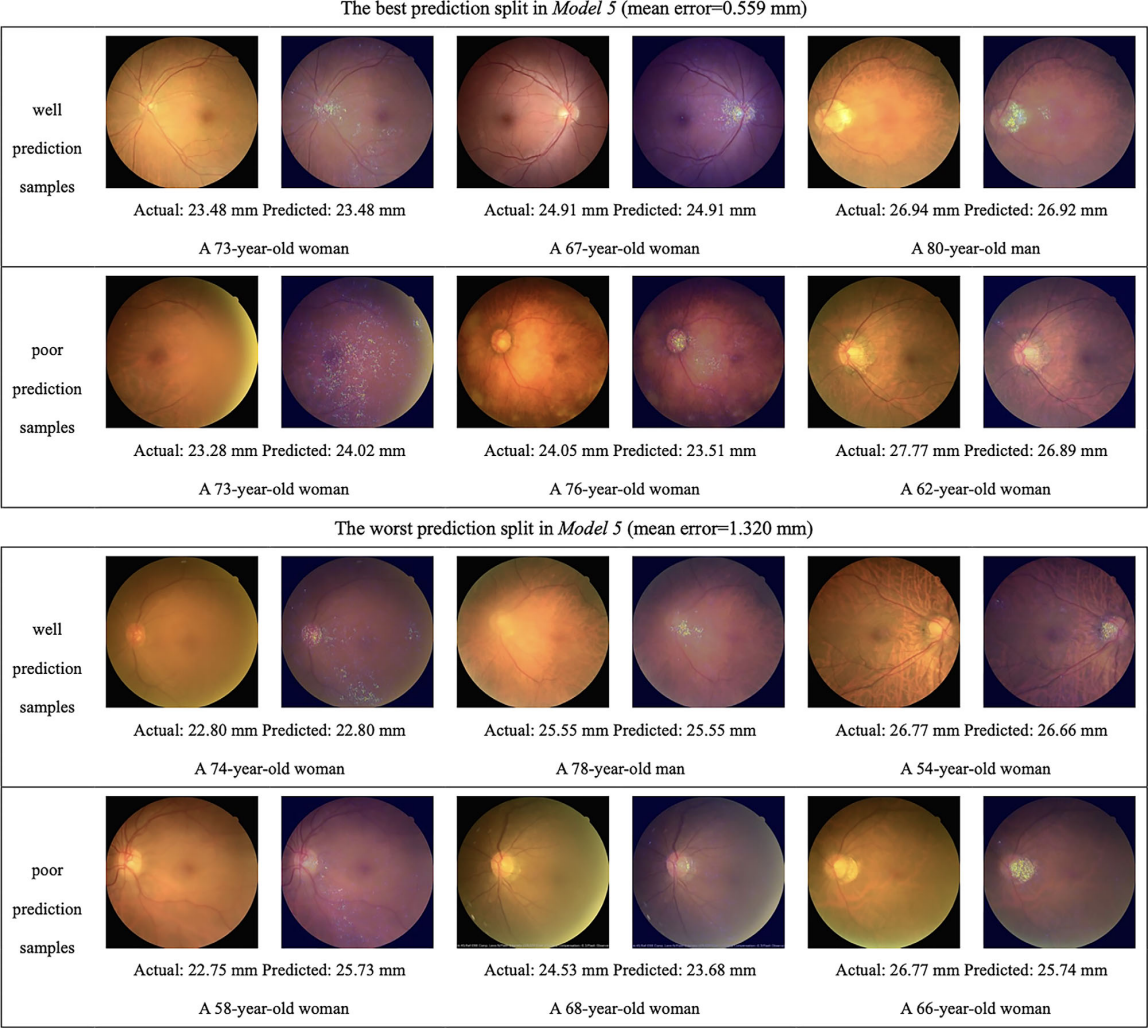
Examples consist of 24 eyes’ CFP from the best and worst prediction split between models 5 and 6 and their heatmaps of integrated gradients, which are on the *right* of the raw images. Heatmaps generated from integrated gradients indicated the area highly recognized by deep learning models, which are seen as scattered bright pixels.

**Figure 4.**
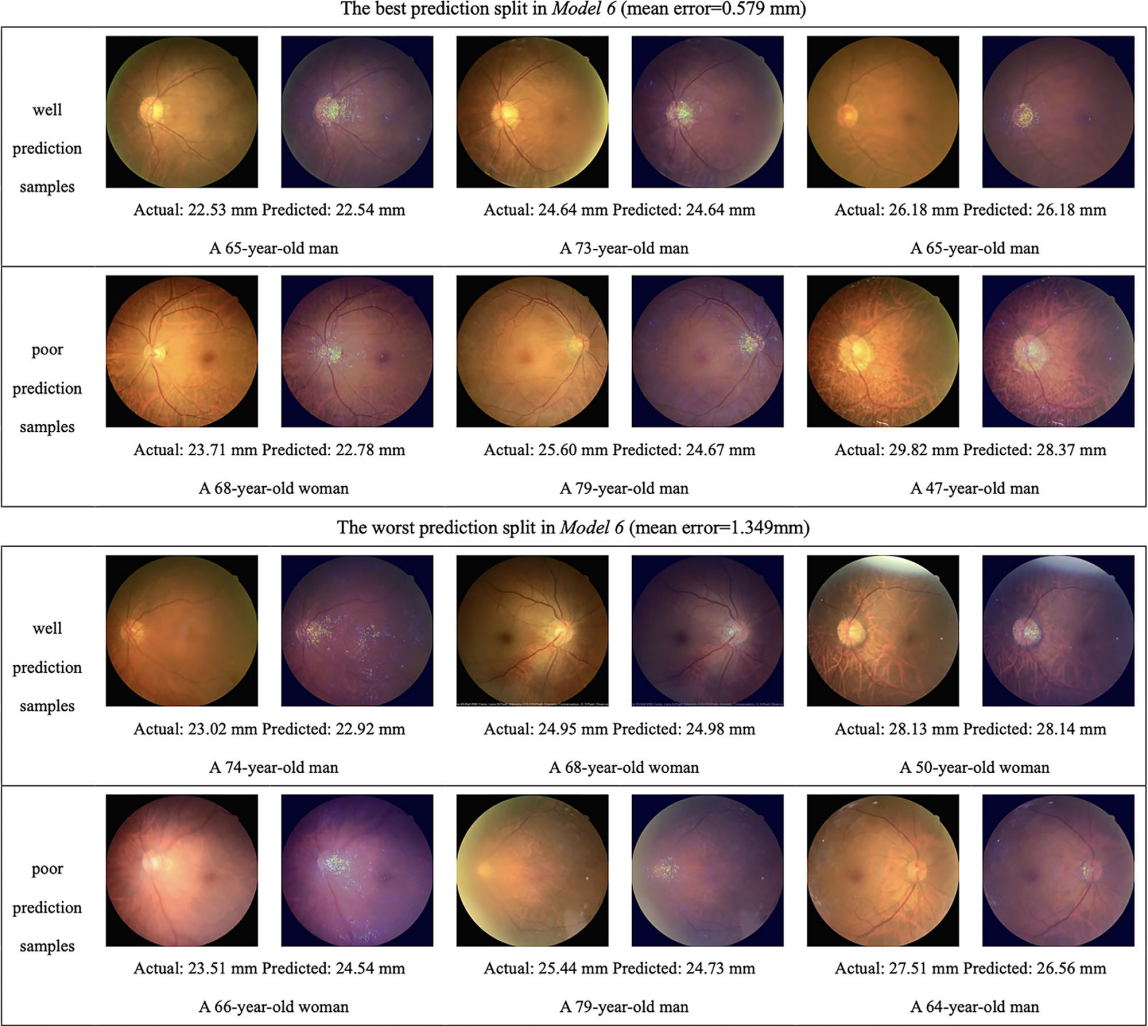
Continued

## Discussion

In the present study, our deep learning models for AL prediction using CFP achieved a mean MAE of 0.759 mm in eyes, with ALs ranging from 19.91 to 32.59 mm. Dong et al.^6^ reported a mean MAE of 0.56 mm for their deep learning model, which included participants aged 50 years or older, and the cohort had mean ± SD AL = 23.24 ± 1.15 mm and a low proportion of long eyes (3.1% of eyes AL ≥26 mm). Considering the longer mean AL (mean ± SD AL = 24.80 ± 1.99 mm) and the much higher proportion of highly myopic eyes (Table 1, 24.2% of eyes AL ≥26 mm) in our cohort, the performance of our models is likely comparable to that reported by Dong et al.^6^ Furthermore, we also found that the inclusion of age as a predictor, in addition to CFP, improved the accuracy of AL prediction. To the best of our knowledge, this is a novel finding that has not been shown previously.

AL and age were well-established factors that can lead to visible changes in the fundus. Regarding the AL, elongated eyeballs could cause a decrease in microvasculature density in the macula,^20^ visible fundus tessellations,^21^ and deep learning detectable changes in choroidal thickness.^22^ In addition, various structural abnormalities involving the optic disc were found associated with elongated AL, including PPA,^23^ tilted disc, and enlarged optic disc crescent.^24^^,^^25^ On the other hand, aging could affect the appearance of the fundus captured by CFP with multiple means, which include influencing the color presented in the photos, altering the retinal microvasculature, increasing the visibility of large choroidal vessels due to tessellation,^11^^,^^26^ and even causing age-related macular degeneration.^27^ In addition to AL and age, Korot et al.^8^ demonstrated that CFP could be used to predict sex via deep learning models, suggesting the presence of sex-related differences in the appearance of the fundus. Ilanchezian et al.^28^ found that the optic disc and the macula were most relevant in fundus-based prediction of sex, with the former providing information on predicting the male sex and the latter on predicting the female sex. Therefore, to more accurately predict AL using CFP, we investigated the effects of adding age and sex as input parameters to the deep learning models and found an improved accuracy mainly attributed to the addition of age as the predictor. This finding suggests that AL and age may share some common characteristics regarding the fundus appearance. Analytical capabilities of CNNs have been proven to extend beyond current human capability, and these CNN models could pick up on both nonhuman-interpretable features and are indicative of meaningful medical information contained in CFP.^29^ The incorporation of age as an additional predictor, while being proved to be advantageous, pointed to the inherent challenge of distinguishing certain specific features solely from the available data, hinting at the potential necessity for supplemental external information. It is crucial to emphasize that the finding needs further substantiation through additional pathologic evidence; nevertheless, it uncovered a noteworthy morphologic observation and provided a clear trajectory for prospective research endeavors.

Based on the heatmap analysis, the most relevant structural characteristics for predicting AL were the optic disc and temporal peripapillary area, followed by tessellation from the choroidal vessels. Disc–fovea distance, disc area, and tessellations have been found to be associated with AL in prior studies,^30^^–^^32^ which supports our results. Notably, both aging and eyeball elongation may result in fundus tessellation and retinal microvasculature change, such as decreased microvessel density, while disc tilting and PPA usually appear only in eyes with elongated AL but not aging eyes. This may explain the greater importance of the optic disc and peripapillary area as compared to tessellated choroidal vessels in AL prediction when age was adjusted. Interestingly, in images that resulted in poor model prediction, the optic disc and peripapillary area remained more frequently highlighted. The deep learning models tended to overestimate AL in eyes with a shorter AL or a larger temporal PPA. The characteristics of sex differences in the optic disc should be different from the morphologies associated with the AL. Thus, whether sex is included as an input parameter or not, it will not interfere with the prediction of AL using CFP. Furthermore, CFPs included in this study were sampled from preoperative examinations for cataract surgeries. Although we cannot conclude on the effect of cataracts on AL prediction, since the CFPs obtained from eyes with severe cataracts and poor visibility of retinal vessels on the photos were excluded from this study, our results suggested that mild to moderate cataracts did not significantly affect the models’ performances. Of note, based on the result of no significant difference between models 5 and 6, we could conclude that once the CFP fulfilled the criteria of the clearly visible optic disc and the central fovea, the image quality would not have a great effect on the output performance.

The MAE of the best-performing model in our study was 0.759 ± 0.120 mm, which may help in evaluating axial myopia. However, for the preoperative calculation of intraocular lens power, a 1-mm error in AL may result in an error as great as 2.4 to 3 diopter in postoperative refraction.^33^^,^^34^ Thus, it might not be practical to use the deep learning model for this task. Nevertheless, the deep learning models may assist in fundus defect differentials during the retinal examination. This means when there is a significant discrepancy between deep learning model-predicted AL and optical biometers-measured AL, the possible presence of structural abnormalities in the fundus should be considered.

This study has some limitations. First, our study cohort was of a single racial/ethnic background. Therefore, whether our results can be extrapolated to other ethnic/radical populations is unknown.^35^^,^^36^ Similarly, whether our results can be generalized to populations outside of the 39- to 92-year age range remains unclear. Second, we included macula- and optic disc–centered photos. Although this could increase the sample size of training data, such data heterogeneity may affect the model performance and increase prediction errors, as reported previously.^6^ Third, the sample size in our study was much smaller than that in Dong et al.^6^ (1105 vs. 5688 images in the development set). However, the difference in MAE was not as significant (0.77 vs. 0.56 mm), suggesting our models were well trained.

In conclusion, this study demonstrated that deep learning–based models could predict AL with moderate accuracy using CFP, and the addition of age as an input parameter can further improve the model performance. Moreover, the optic disc and temporal peripapillary areas may contain structural information most relevant to the prediction of AL.
